# Structure–activity relationship of synthetic cathinones: integrated in silico, in vitro, and in vivo studies of α-PiHP analogues

**DOI:** 10.1007/s00204-026-04483-z

**Published:** 2026-06-27

**Authors:** Martalu D. Pazos, Guillermo García-Díez, David Pubill, Xavier Berzosa, Roger Estrada-Tejedor, Jorge Camarasa, Elena Escubedo, Raúl López-Arnau, Núria Nadal-Gratacós

**Affiliations:** 1 Department of Pharmacology, Toxicology and Therapeutic Chemistry, Pharmacology Section, Institute of Biomedicine (IBUB), Faculty of Pharmacy and Food Science, Universitat de Barcelona, 08028 Barcelona, Spain; 2 Chemical Reactions for Innovative Solutions (CRISOL), IQS School of Engineering, Universitat Ramon Llull, 08017 Barcelona, Spain; 3 Department of Pharmaceutical Chemistry, IQS School of Engineering, Universitat Ramon Llull, 08017 Barcelona, Spain

**Keywords:** New psychoactive substances (NPS), Synthetic cathinones, Structure–activity relationship (SAR), α-PiHP, MDPiHP

## Abstract

Synthetic cathinones represent the main group of emerging psychostimulants consumed worldwide. Structural modifications of these compounds have led to pyrovalerones, potent dopamine transporter (DAT) inhibitors associated with high abuse liability. Among them, α-pyrrolidinoisohexanophenone (α-PiHP) became the most seized pyrovalerone in the European Union in 2023. Novel analogues have rapidly appeared, yet their neuropharmacological and toxicological properties remain unknown. This study aimed to characterize α-PiHP and five structurally related derivatives (3-Me-α-PiHP, 4-Me-α-PiHP, 3-F-α-PiHP, 4-F-α-PiHP, and MDPiHP). Monoamine transporter activity was evaluated using HEK293 cells expressing human norepinephrine (hNET), hDAT, or serotonin (hSERT) transporters through uptake inhibition and binding assays. Molecular docking was performed to model interactions at hDAT. Psychostimulant, thigmotactic, and rewarding effects were evaluated in male Swiss CD-1 mice using horizontal locomotor activity, open field, and conditioned place preference paradigms. Lethality after drug administration was also assessed. All compounds potently inhibited hDAT and hNET, while displaying limited activity at hSERT. This resulted in high hDAT/hSERT ratios indicative of a stimulant-like profile and elevated abuse liability, confirmed in vivo by pronounced psychostimulant and rewarding effects. All molecules interacted with the orthosteric binding site at hDAT and displayed higher binding affinity than cocaine in in vitro and in silico studies. *Meta*-substituted analogues showed greater hDAT affinity and behavioural responses than *para*-isomers. Notably, MDPiHP exhibited the highest potency and affinity, strongest psychostimulant effects and significant lethality. These findings demonstrate that minor structural modifications markedly influence pharmacological activity of pyrovalerone derivatives, highlighting the high abuse potential and health risks associated with emerging α-PiHP analogues.

**Supplementary Information:**

The online version contains supplementary material available at https://doi.org/10.1007/s00204-026-04483-z.

## Introduction:

New Psychoactive Substances (NPS) are drugs of abuse designed to mimic the effects of illegal drugs such as cocaine, MDMA, or methamphetamine while evading the existing legislation and drug testing (United Nations Office on Drugs and Crime 2013). Their online availability, relatively low cost, ambiguous legal status, and effects comparable to controlled substances have facilitated a shift from traditional drugs to NPS (European Union Drugs Agency 2025), fostering a false sense of safety among users. These factors, combined with limited access to conventional adulterants, make NPS attractive to dealers, who often use them as replacements of traditional drugs and fillers to increase profits (Oliver et al. 2019; Chen et al. 2024). Moreover, once a derivative is placed under control, new unregulated analogues can be readily synthesized to replace it. As a result, data on their toxicity and abuse potential are often unavailable or severely limited, while the long-term adverse effects remain largely unknown, thereby posing a significant public health threat (European Union Drugs Agency 2025).

Among NPS, synthetic cathinones (SCs) represent the main group of emerging psychostimulants currently consumed worldwide. SCs are β-ketone amphetamine derivatives of cathinone, an active alkaloid found in the *Catha edulis* shrub (Nadal-Gratacós et al. 2024; European Union Drugs Agency 2025). The pharmacological action of SCs involves modulation of monoamine signalling. SCs increase synaptic monoamine levels through monoamine transporter blocking or by inducing monoamine release. In this scenario, pyrovalerone derivatives—SCs containing N- pyrrolidinyl substitution—act primarily as transporter blockers, with high selectivity for the dopamine (DA) transporter (DAT) over the serotonin (5-HT) transporter (SERT), resulting in potent psychostimulant and reinforcing effects (Rickli et al. 2015; Gannon et al. 2018; Kolaczynska et al. 2021). Representative compounds include α-pyrrolidinopentiophenone (α-PVP, “*flakka*”) and methylenedioxypyrovalerone (MDPV, “*Monkey Dust*”), both showing long-lasting psychomotor stimulant, rewarding, and reinforcing properties in rodents (Aarde et al. 2013 and 2015; Karlsson et al. 2014; Gatch et al. 2015). Due to their severe adverse effects and widespread media attention, both were included in Schedule II of the 1971 Convention in 2015 and 2016 (Commission on Narcotic Drugs 2015 and 2016). Following their control, new analogues rapidly emerged, such as α-pyrrolidinohexanophenone (α-PHP) and its 3,4-methylenedioxy analogue (MDPHP), which maintain high affinity for DAT and the norepinephrine (NE) transporter (NET), as well as potent psychostimulant effects (Kolanos et al. 2015; Eshleman et al. 2017 and 2018; Kolaczynska et al. 2021). α-PHP was placed in schedule II on the list of Psychotropic Substances under International Control in 2020 (Commission on Narcotic Drugs 2020).

α-Pyrrolidinoisohexanophenone (α-PiHP), an α-PHP chain isomer, was first identified in 2016 and by 2023 became the most seized pyrovalerone in the EU (1.46 tonnes), reported in at least 30 countries (World Health Organization 2023; European Union Drugs Agency 2025). In vitro*,* α-PiHP is a potent DAT inhibitor, with potency comparable to that of α-PVP and a high DAT/SERT ratio (Persson et al. 2024; Baarset et al. 2025), suggestive of a high abuse potential. In vivo studies have shown that α-PiHP produces robust psychostimulant effects and fully substitutes for the discriminative stimulus effects of cocaine and methamphetamine, indicating similar subjective-like effects in animal models (Shetty et al. 2023). Reported effects in humans include euphoria and stimulation; while adverse effects included tachycardia, hyperthermia, vasoconstriction, paranoia and aggression (Brueckner et al. 2024; T’kind et al. 2024). Due to its rapid spread, it was placed in Schedule II on the list of Psychotropic Substances under International Control in 2023 (Commission on Narcotic Drugs 2023).

Given the continued emergence of structurally related cathinones following international scheduling, it is critical to identify and characterize potential α-PiHP replacement candidates and to elucidate the SAR governing their neuropharmacological and toxicological profiles. In this context, ring-substitutions such as methyl-, fluoro-, and methylenedioxy- groups are among the most frequently observed modifications in emerging cathinone analogues (Kelly 2011; Fornal 2013). Accordingly, several α-PiHP derivatives bearing these substituents have recently appeared on the drug market. For instance, 4-F-α-PiHP, 3-F-α-PiHP (4-Fluoro-α-pyrrolidinoisohexanophenone and 3-Fluoro-α-pyrrolidinoisohexanophenone, respectively) and MDPiHP (3,4-methylenedioxy-α-pyrrolidinoisohexanophenone) were first reported between 2017 and 2020 (European Monitoring Centre for Drugs and Drug Addiction 2017, 2020); while methyl-substituted analogues appeared more recently (European Monitoring Centre for Drugs and Drug Addiction 2022). Several forensic toxicological cases involving MDPiHP, 3-Me-α-PiHP (3-methyl-α-pyrrolidinoisohexanophenone) and 4-Me-α-PiHP (4-methyl-α-pyrrolidinoisohexanophenone) have been reported since 2020, including two fatalities associated with 3-Me-α-PiHP in which co-ingested substances were primary contributors to death (Green et al. 2025). Moreover, an adulteration case involving substitution of 4‐fluoromethylphenidate (4F-MPH) with 4-F-α-PiHP was reported in 2023 (Algar et al. 2024). However, to date, little is known regarding the in vitro*, *in silico, and/or in vivo neuropharmacology of these novel SCs.

**Fig. 1:**
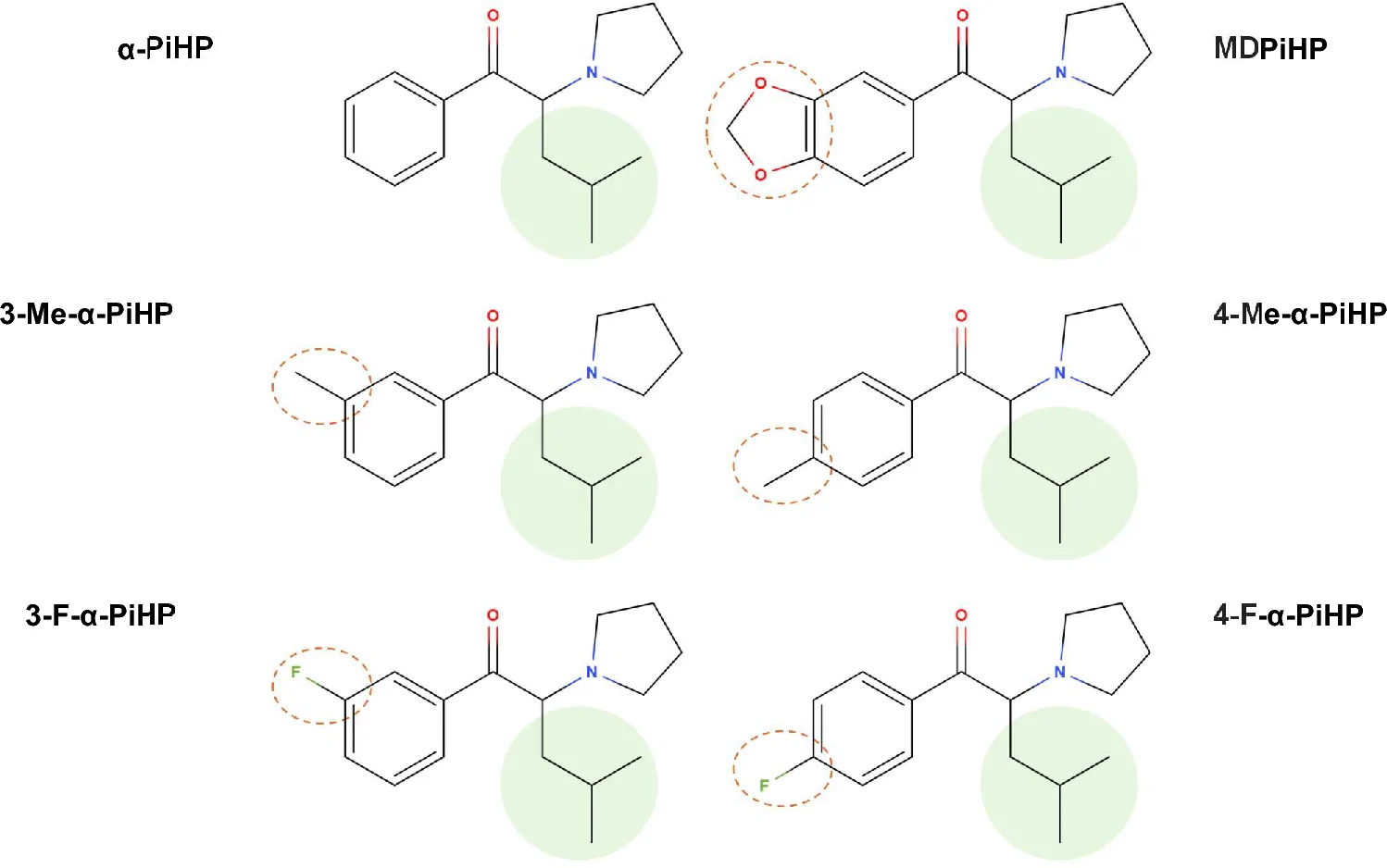
Chemical structure of α-PiHP, MDPiHP, 3-Me-α-PiHP, 4-Me-α-PiHP, 3-F-α-PiHP and 4-F-α-PiHP

Therefore, the aim of this study was to comprehensively investigate α-PiHP and five of its analogues, namely MDPiHP, 3-Me-α-PiHP, 4-Me-α-PiHP, 3-F-α-PiHP, and 4-F-α-PiHP (Fig. 1) by: (i) elucidating their mechanism of action through monoamine uptake inhibition, transporter binding assays, and molecular docking studies; and (ii) assessing their in vivo psychostimulant and rewarding properties in male mice. This dual approach was designed to provide detailed insights into their Structure–Activity Relationship (SAR), establishing a link between molecular interactions and behavioural effects. Ultimately, these data are intended to provide scientifically grounded evidence to support risk assessment and enhance preparedness for potential future emergencies involving emerging SCs.

## Materials and methods

### Subjects

Male Swiss CD-1 mice (Janvier, Le Genest, France) weighing 30–40 g (6–8 weeks old) were randomly assigned to an experimental group. The animals were housed (seven per cage) in temperature-controlled conditions (22 ± 1 °C) under a 12 h light/dark cycle and had free access to food and drinking water. All the behavioural experiments were performed during the mice’s light cycle. The animals were supervised immediately after injection and during the behavioural procedure, and different parameters were visually evaluated for humane endpoints, such as self-mutilations, abnormal posture, or a weight loss greater than 20%. No overt signs suggestive of sensitization or tolerance affecting behavioural performance were observed during the study. The same cohort of animals was used in the locomotor activity and conditioned place preference (CPP) experiments in order to reduce the total number of animals used. The experimental sessions were spaced apart by appropriate intervals to minimize carryover effects. All animal care and experimental protocols were approved by the Animal Ethics Committee of the Universitat de Barcelona and were in accordance with the guidelines of the European Community Council (2010/63/EU), as amended by Regulation (EU) 2019/1010, and the ARRIVE guidelines for reporting experiments involving animals (Percie du Sert et al. 2020). Project code:110/22.

### Drugs and reagents

α-PiHP and its derivatives were synthetized in racemic form as hydrochloride salts following the procedure described by (Meltzer et al. 2006) with minor modifications; for further details see Supplemental Material. Solutions for injection were prepared daily in isotonic saline solution (0.9% NaCl, pH 7.4). All administered doses refer to the hydrochloride salt form. [^3^H]1-Methyl-4-phenylpyridinium ([^3^H]MPP +), [^3^H]5-HT and [^3^H]WIN35.428 were purchased from Revvity Inc. (Boston, MA, USA). Cell culture media (Dulbecco’s Modified Eagle’s medium (DMEM) high-glucose) and HEPES sodium were purchased from Sigma-Aldrich. The Spanish National Institute of Toxicology provided cocaine. All other reagents were of analytical grade and purchased from several commercial sources.

### Monoamine uptake inhibition and DAT binding assays in HEK293 cells

#### Cell culture and membrane preparation

Human embryonic kidney (HEK293) cells (CLS Cat# 300,192/p777_HEK293, RRID: CVCL_0045) stably transfected with the human isoforms of the NET, DAT and SERT (hNET, hDAT and hSERT) were used for the uptake inhibition and transporter binding assays. The generation and maintenance of stable, monoclonal cell lines expressing hNET, hDAT and hSERT were conducted as described by (Mayer et al. 2016). Cells were maintained in DMEM supplemented with heat-inactivated 10% foetal bovine serum (FBS), 1 μg/mL streptomycin, and 100 U/mL penicillin at 37 °C in a 5% CO2 humidified atmosphere. Geneticin (G418; 50 μg/mL) was added to maintain the selection process. Upon approximately 80% confluence, cells were washed with 5 mL of phosphate-buffered saline (PBS) and 1 mL of trypsin/EDTA was added. After 3 min, 9 mL of DMEM was added to stop cell trypsinization. For the uptake inhibition assays, cells were then resuspended, counted, and seeded (0.18 MiO/mL density) onto 96-well plates previously treated (24 h) with poly-D-lysine (PDL).

For membrane preparations, stably transfected hDAT HEK293 cells were harvested from dishes 80–90% confluent. Briefly, cells were washed twice with ice-cold phosphate buffered saline (PBS), mechanically detached from the dish with a plastic scraper in the same ice-cold PBS and pelleted by centrifugation (400 × g for 10 min at 4ºC). The resulting pellet was resuspended in HME buffer (20 mM HEPES NaOH, 2 mM MgCl_2_, 1 mM EDTA; pH 7.4), followed by two freeze–thaw cycles in liquid nitrogen and homogenization through sonication at 4ºC. Membranes were collected by centrifugation (40.000 × g for 30 min at 4ºC) and resuspended in an appropriate volume of HME buffer. Protein concentration was determined using the Bradford assay, with the Bio-Rad Protein Dye Reagent (Bio-Rad Laboratories, Hercules, CA). The membrane preparations were stored at − 80ºC. For both uptake inhibition assays and transporter binding assays, α-PVP, MDPV and cocaine were used as reference drugs.

#### Uptake inhibition assays

Before initiating the uptake inhibition assay, the culture medium in the 96-well plates was aspirated and immediately replaced with 200 µL of Krebs-HEPES buffer (KHB; 10 mM HEPES, 120 mM NaCl, 3 mM KCl, 2 mM CaCl_2_, 2 mM MgCl_2_with 20 mM D-glucose; pH 7.3). A 5-min preincubation phase was then carried out, in which the cells expressing hNET, hDAT and hSERT were exposed to different concentrations of the cathinone -from 0.01 nM to 30 mM- in KHB at a final volume of 50 µL/well. Following removal of the preincubation solution, cells were incubated with the radiolabelled substrates- 0.02 µM [^3^H]MPP⁺ for 3 min (hNET and hDAT), and 0.1 µM [^3^H]5-HT for 1 min (hSERT)- in the presence of the corresponding drug concentrations in KHB. At the end of the incubation period, the uptake solution was aspirated, and the cells were washed with ice-cold KHB to stop further uptake. Finally, cells were lysed with 1% sodium dodecyl sulphate (SDS), and the lysates were mixed with scintillation fluid for quantification of radioactivity using a beta-scintillation counter (PerkinElmer, Waltham, MA, USA). Non-specific uptake was assessed in parallel samples with 100 µM cocaine (for hNET and hDAT) or 30 µM paroxetine (for hSERT) to ensure complete inhibition. The non-specific uptake value was less than 10% of the total uptake and was subtracted from the data to determine specific uptake. Uptake in the absence of drug was normalized to 100%, with uptake in the presence of varying drug concentrations expressed as a percentage of this value. Four independent experiments were conducted, each performed in triplicate.

#### hDAT binding assays

Each cathinone was dissolved in binding buffer (120 mM NaCl, 3 mM KCl, 10 μM ZnCl_2_, 20 mM Tris and 2 mM MgCl_2_, pH 7.4) at a range of concentrations from 0.1 nM to 100 µM. 15 µg of membranes overexpressing the hDAT transporter in binding buffer were incubated with 25 μL of [^3^H]WIN35,428 (Kd = 12 nM; Bmax = 6.75 pmol/ mg) (Sucic et al. 2010) and 125 μL of the diluted cathinone. Non-specific binding was assessed in parallel samples with 100 µM cocaine to ensure complete radioligand displacement.

Incubation was carried out for 1 h at 22 °C. Binding reactions were stopped by filtration of the membranes through GF/C glass microfiber filters pre-soaked in 0.5% polyethyleneimine, followed by washing with ice-cold wash buffer (120 mM NaCl, 2 mM MgCl₂, 10 mM Tris, and 100 μM ZnCl₂, pH 7.4). The filters were transferred to scintillation vials with scintillation liquid, and the retained radioactivity was measured. The non-specific binding value was less than 10% of the total displacement and was subtracted from the data to determine specific binding. Binding in the absence of drug was normalized to 100%, with the specific binding of different drug concentrations to hDAT expressed as a percentage of this value. Four independent experiments were conducted, each performed in duplicate.

### Molecular docking at hDAT

All molecular docking studies were done employing the software Molecular Operating Environment (MOE), 2024.06 Chemical Computing Group ULC, 910–1010 Sherbrooke St. W., Montreal, QC H3A 2R7, 2025. The crystal structure of the human dopamine transporter (hDAT) was obtained from PDB (ID: 9EO4). This crystal structure was published in 2024, including the wildtype hDAT bound to cocaine at 2.66 Å resolution (Nielsen et al. 2024). Molecular docking protocol was validated using the cocaine molecule (included in the PDB complex) as reference. Guided docking was applied considering the cocaine’s binding cavity. Water molecules, ions and lipidic molecules were removed, except for the three structural water molecules close to the benzene ring in cocaine (Nielsen et al. 2024). The molecular system was prepared according to the Structure Preparation module of MOE and hydrogen atoms were added by means of 3D Protonate option. The Triangle Matcher method was employed to carry out the Placement, and the London dG scoring function was used. A total of 100 poses were allowed to pass to the Refinement step, considering Rigid Receptor Model, keeping the scoring function, obtaining a final set of 5 poses. This methodology was validated yielding a pose which mimicked the experimental pose of the cocaine molecule, with an RMSD of 0.54 Å.

After validating the docking procedure, the six cathinones under study were modelled in MOE. Only the (S) enantiomers were considered, given that, generally, for cathinones this is the eutomer (Niello et al. 2023). The protonation state at pH = 7 was set using the Wash tool in MOE. As expected, all cathinones are protonated at this pH. An energy minimization calculation was also conducted in MMFF94x forcefield, using the default settings.

### Horizontal locomotor activity and thigmotaxis

The psychostimulant effect was studied through the horizontal locomotor activity (HLA) test. First, a habituation phase was performed to reduce the novelty and stress associated with handling and injection. During this phase, mice received an intraperitoneal (i.p.) saline injection and were placed into a black Plexiglass open field (OF) arena (25 × 25 × 40 cm) under low-light conditions and white noise for 30 min, for two consecutive days. On the test day, the animals received an i.p. injection (5 mL/kg) of saline or different doses (2.5, 10, or 25 mg/kg) of the corresponding drug (α-PiHP, MDPiHP, 3-Me-α-PiHP, 4-Me-α-PiHP, 3-F-α-PiHP and 4-F-α-PiHP) and were immediately placed in the OF arena with the same conditions of light and noise. The HLA was monitored for 1 h, and their total travelled distance (in cm) was measured using a specific tracking software (Smart 3.0 Panlab, Barcelona, Spain). Moreover, thigmotaxis defined as the tendency of rodents to remain close to the walls of the arena and commonly interpreted as an indicator of anxiety-like behaviour, was also assessed (Simon et al. 1994). The time spent, in seconds, in the centre (8 × 8 cm) or the periphery of the arena was monitored for 60 min (Smart 3.0 Panlab, Barcelona, Spain). The doses were chosen according to the psychostimulant effect induced by structurally related SCs published in previous studies by our research group (Nadal-Gratacós et al. 2022).

### Conditioned place preference

The potential of α-PiHP and its derivatives to induce rewarding effects was assessed using the CPP paradigm. The device consisted of two compartments, distinguished by different tactile and visual features, and connected by a central corridor. The test was performed in three phases: preconditioning, conditioning and post-conditioning phase. During the preconditioning phase, mice had free access to both compartments for 15 min, and the time spent in each compartment was recorded and monitored (Smart 3.0 Panlab, Barcelona, Spain). During the conditioning phase (8 sessions; 2 sessions per day), animals were treated with an i.p. injection of the corresponding molecule and were immediately placed into one of the compartments (drug-paired compartment) for 20 min. The doses tested were the same used in the HLA experiment. On alternate sessions, the animals received saline and were confined to the opposite compartment (drug-free compartment) for 20 min. The compartment and session in which mice received the drug were randomized. Mice in the control group received a saline injection in every session. The postconditioning phase was conducted exactly as in the preconditioning phase; animals were drug-free, had free access to both compartments for 15 min and the time spent in each compartment was monitored. A preference score was calculated for each animal as the difference between the time spent in the drug-paired compartment during the post-conditioning phase and in the preconditioning phase. An exclusion criterion was predefined for animals that had an initial preference for one of the compartments (> 70% of the total session time) in the preconditioning phase.

### Data and statistical analysis

Sample size was determined using GPower software, with a significance level (α) of 0.05 and statistical power of 0.8. Data are presented as mean ± SEM. Nonlinear regression was applied to fit sigmoidal dose–response curves for α-PiHP, 3-Me-α-PiHP, 4-Me-α-PiHP, 3-F-α-PiHP, 4-F-α-PiHP, MDPiHP, α-PVP, MDPV, and cocaine to calculate IC₅₀ values (μM). The Cheng–Prusoff equation was used to determine *K*_i_ values (nM): *K*_i_ = EC_50_/ (1 + [radioligand concentration/ *K*_d_]) (Yung-Chi and Prusoff 1973). DAT/SERT inhibition ratios were calculated as 1/DAT IC_50_:1/SERT IC_50_. For the order of potencies Kruskall-Wallis or one-way ANOVA followed by Dunn’s or Tukey’s multiple comparisons test was used. For in vivo experiments, Kruskall-Wallis, one-way or two-way repeated measures ANOVA followed by Dunn’s or Tukey’s multiple comparisons test was used to assess the effects of α-PiHP and its derivatives. The α-error probability was set at 0.05 (*p* < 0.05). All statistical analyses were performed using GraphPad Prism (GraphPad Software, San Diego, CA, USA). Complete statistical results are provided in Supplemental Material.

## Results

### α-PiHP analogues are potent hNET and hDAT uptake inhibitors with limited hSERT activity

**Fig. 2:**
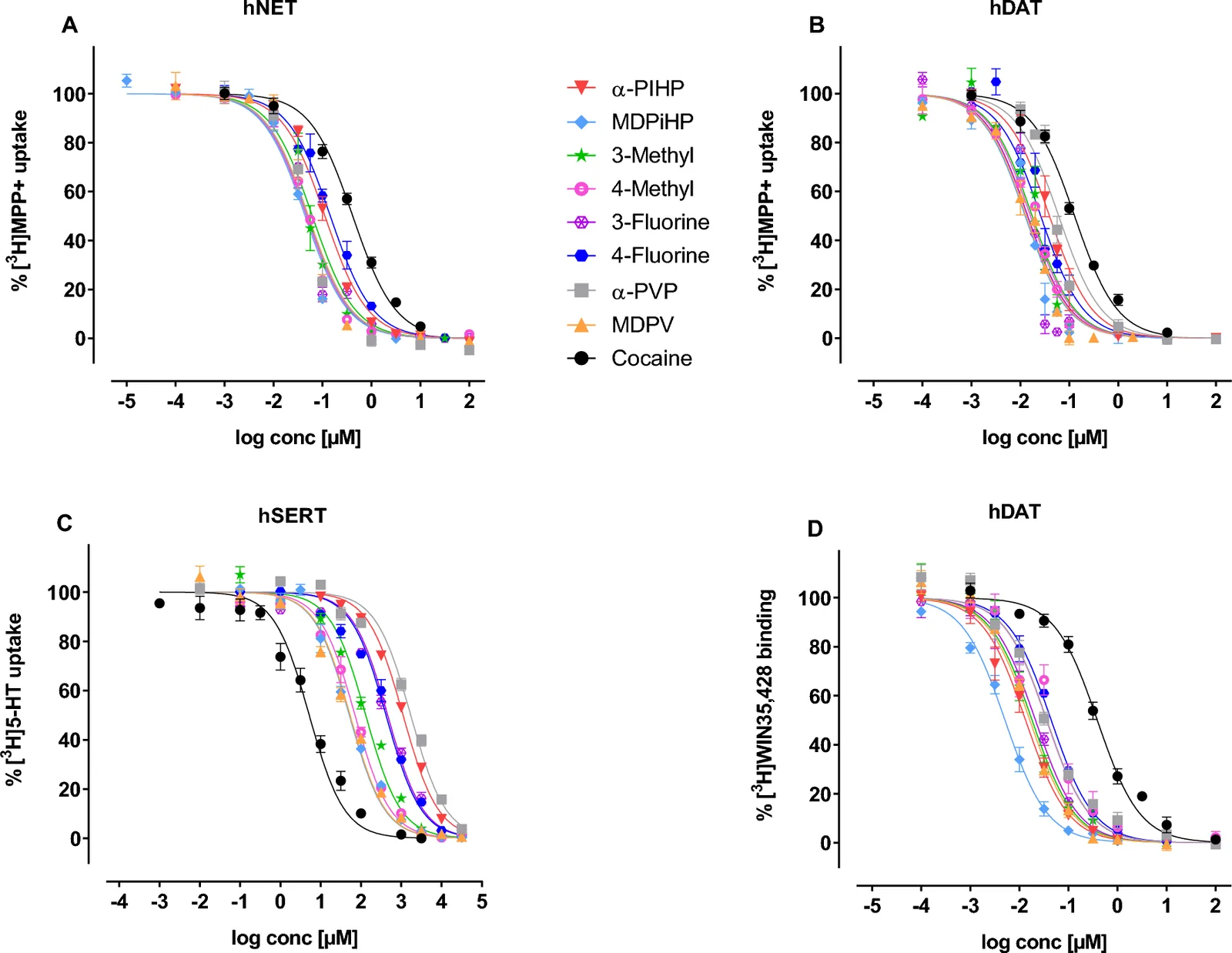
Concentration-effect curves of α-PiHP, 3-Me-α-PiHP, 4-Me-α-PiHP, 3-F-α-PiHP, 4-F-α-PiHP, MDPiHP, α-PVP, MDPV and cocaine on [^3^H]MPP + uptake at hNET and hDAT (**A**–**B**), [^3^H]5-HT uptake at hSERT (**C**) and [^3^H]WIN35,428 binding at hDAT (**D**) in transfected HEK293 cells. Data are expressed as a percentage of control uptake or binding (mean ± SEM) of four independent experiments performed per triplicate

The inhibitory potencies of the tested compounds at the monoamine transporters are expressed as IC₅₀ values and are summarised in Table 1 together with the hDAT/hSERT inhibition ratios. The concentration–response curves are presented in Fig. 2A–C.

**Table 1:** Monoamine uptake inhibition at hNET, hDAT and hSERT and transporter binding affinities at hDAT of substituted cathinones and cocaine

Transfected HEK293 Cells						
Drug	IC_50_; Monoamine Uptake Inhibition (μM)			*K*_i_; Transporter Binding Affinities (μM)		
	[^3^H]MPP +		[^3^H]5-HT	hDAT/hSERT Inhibition Ratio	[^3^H]WIN35,428	*K*_i_ [^3^H]WIN35,428/ IC_50_[^3^H]MPP + Ratio
	hNET	hDAT	hSERT		hDAT	
α-PiHP	0.111 (± 0.011)	0.044 (± 0.009)	> 100	> 10,000	0.009 (± 0.002)	0.197
MDPiHP	0.044 (± 0.003)	0.013 (± 0.002)	53.29 (± 4.82)	4080	0.003 (± 0.001)	0.229
3-Me-α-PiHP	0.063 (± 0.011)	0.017 (± 0.001)	> 100	7614	0.010 (± 0.001)	0.595
4-Me-α-PiHP	0.047 (± 0.001)	0.018 (± 0.001)	73.71 (± 4.56)	4118	0.025 (± 0.005)	1.375
3-F-α-PiHP	0.046 (± 0.006)	0.014 (± 0.002)	> 100	> 10,000	0.012 (± 0.003)	0.836
4-F-α-PiHP	0.156 (± 0.014)	0.026 (± 0.005)	> 100	> 10,000	0.024(± 0.002)	0.946
α-PVP	0.049 (± 0.003)	0.060 (± 0.002)	> 100	> 10,000	0.019 (± 0.002)	0.326
MDPV	0.055 (± 0.009)	0.015 (± 0.001)	55.43 (± 4.98)	3780	0.009 (± 0.001)	0.620
Cocaine	0.426 (± 0.037)	0.136 (± 0.008)	5.591 (± 1.163)	41	0.213 (± 0.036)	1.564

hDAT/hSERT ratio = 1/hDAT IC_50_: 1/hSERT IC_50_

For monoamine uptake inhibition assays, values are IC_50_, given as μM (mean ± SEM) and for transporter binding affinities assays, values are *K*_i_, given as μM (mean ± SEM) of four independent experiments, each performed in per triplicate.

All SCs demonstrated significantly lower IC₅₀ values at the hNET than cocaine, indicating a markedly greater inhibitory effect. Among the α-PiHP derivatives, the most potent inhibitors were 3-Me-α-PiHP, 4-Me-α-PiHP, 3-F-α-PiHP, and MDPiHP which exhibited approximately a ten‑fold higher potency than cocaine and showed a similar ability to inhibit uptake at hNET than MDPV and α-PVP. The order of potency at hNET was: MDPiHP≈3-F-α-PiHP≈4-Me-α-PiHP≈α-PVP≈MDPV≈3-Me-α-PiHP≈α-PiHP ≥ 4-F-α-PiHP >  > Cocaine. Complete statistical results are provided in Supplemental Material Table S1.

All compounds were more potent DA uptake inhibitors than cocaine. Consistent with the hNET profile, the most potent hDAT inhibitors were 3-Me-α-PiHP, 4-Me-α-PiHP, 3-F-α-PiHP, and MDPiHP which were up to ten-fold more potent than cocaine, up to four-fold more potent than α-PVP, and showed a similar ability to inhibit uptake at hDAT than MDPV. The order of potency at hDAT was: MDPiHP≈3-F-α-PiHP≈MDPV≈3-Me-α-PiHP≈4-Me-α-PiHP≈4-F-α-PiHP > α-PiHP≈α-PVP >  > Cocaine. Complete statistical results are provided in Supplemental Material Table S1.

Regarding hSERT inhibition, all SCs were weaker inhibitors than cocaine. Among the drugs assessed, those containing the substitution 3,4-methylenedioxy, MDPiHP and MDPV, were the most potent 5-HT uptake inhibitors, though still ten-fold less potent than cocaine. Those containing methyl substitution displayed intermediate activity and fluorine-substituted derivatives were markedly less active. The unsubstituted compounds (α-PiHP and α-PVP) showed the weakest 5-HT inhibition. The order of potency at hSERT was: Cocaine ≫ MDPiHP ≈ MDPV ≈ 4-Me-α-PiHP ≈ 3-Me-α-PiHP > 3-F-α-PiHP ≈ 4-F-α-PiHP > α-PiHP > α-PVP. Complete statistical results are provided in Supplemental Material Table S1. Moreover, all SCs showed higher hDAT/hSERT ratios compared to cocaine.

### α-PiHP analogues show higher binding affinity at hDAT than cocaine

The binding affinity constants of the tested compounds at the hDAT are expressed as *K*_i_ values and are summarised in Table 1. The concentration–response curves are presented in Fig. 2D.

All compounds demonstrated higher affinity for hDAT than cocaine. The highest affinity was observed for MDPiHP, with up to seventy times greater affinity than cocaine. *Meta*-substituted derivatives, 3-Me-α-PiHP and 3-F-α-PiHP, showed *K*_i_ values comparable to those of α-PiHP, while displaying a twofold higher affinity for DAT than their corresponding *para*-substituted derivatives, 4-Me-α-PiHP and 4-F-α-PiHP. The order of affinity for hDAT was: MDPiHP > MDPV ≈ α-PiHP ≈ 3-Methyl-α-PiHP ≈ 3-Fluoro-α-PiHP > α-PVP ≈ 4-Methyl-α-PiHP ≈ 4-Fluoro-α-PiHP ≫ Cocaine. Complete statistical results are provided in Supplemental Material Table S1. Moreover, all pyrrolidine-containing cathinones tested showed a *K*_i_ /IC_50_ uptake inhibition ratio between 0.197 and 1.375.

### α-PiHP analogues exhibit cocaine-like binding at the orthosteric site of DAT

**Fig. 3:**
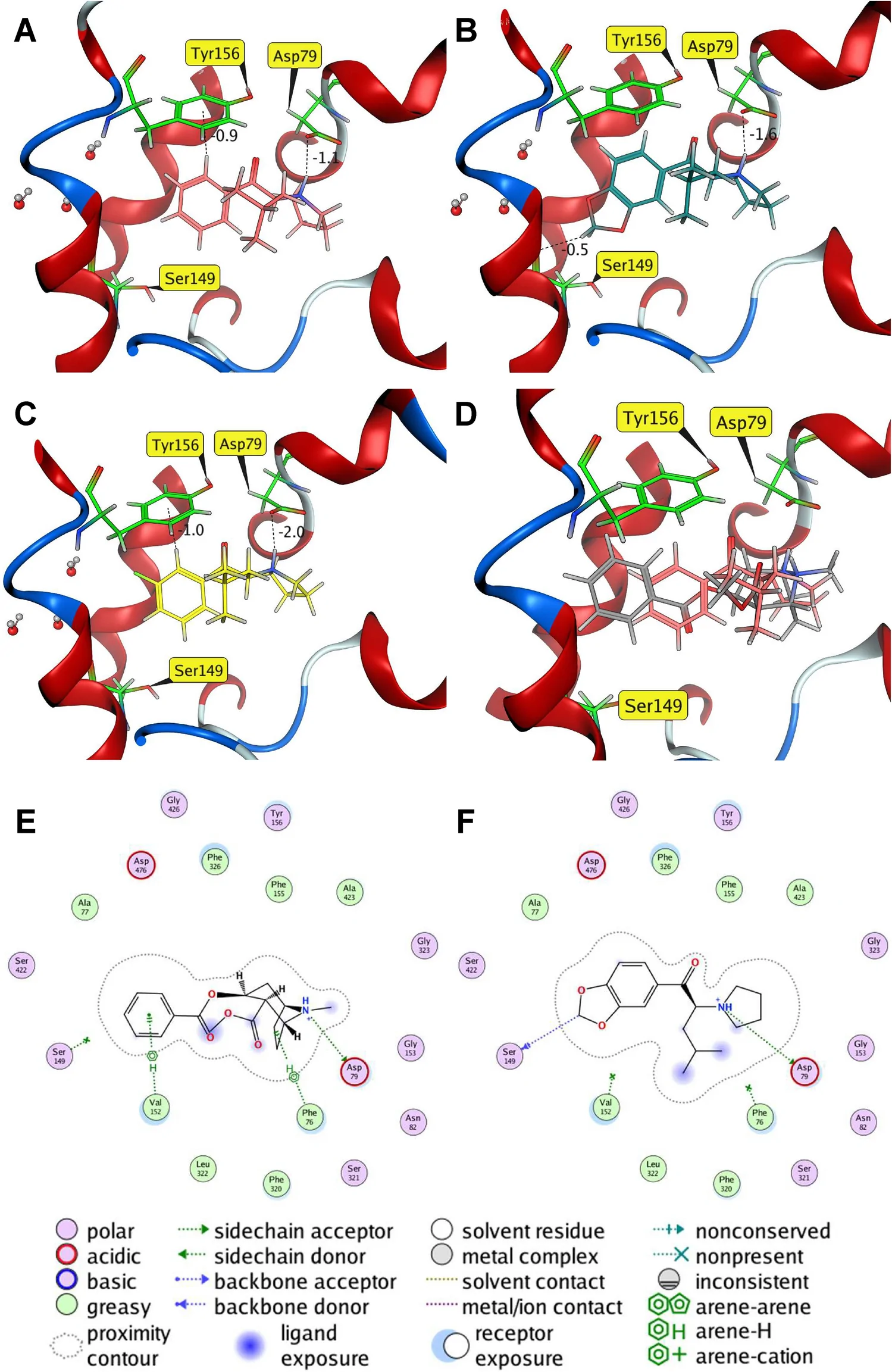
Comparison of the binding interactions of α-PiHP (pink, **A**), MDPiHP (blue,** B**) and 3-F-α-PiHP (yellow, **C**) with DAT. **(D)** Superposition of the binding modes of cocaine (grey) and α-PiHP (pink). (**E, F**) Detailed protein–ligand interaction maps of cocaine and MDPiHP, respectively

All tested cathinones bind in a similar mechanism to the orthosteric site of DAT. Comparing to the crystallized ligand, cocaine, the aromatic ring of all cathinones is displaced to the outside when comparing to cocaine (Fig. 3). The pyrrolidine ring in α-PiHP and its derivatives overlaps with the tropane ring in cocaine, so that the protonated tertiary amine can form a salt bridge with Asp79. Moreover, the isopropyl-containing sidechain coincides with the methyl ester moiety present in the cocaine molecule. Nevertheless, the ketone group in the cathinone molecules is pointing the opposite way to the carbonyl in the benzyl ester group. As shown in Supplemental Material Table S2, the docking scores fall within a narrow range and thus, do not correlate with the *Ki* values. Accordingly, a direct quantitative relationship between predicted ΔG values and experimental *Ki* values is not expected for compounds with similar affinities. Notably, MDPiHP emerges as the most distinct compound, exhibiting a more favourable predicted binding energy, consistent with its higher experimental affinity.

In the case of α-PiHP, the hydrogen at the *ortho* position in the aromatic ring forms an arene-hydrogen interaction with Tyr156, with a strength of -0.9 kcal/mol. Concerning MDPiHP, the salt bridge with Asp79 is conserved, but the arene-hydrogen interaction with Tyr156 is lost. Nonetheless, a novel interaction between the carbonyl group of Ser149 and the methylenedioxy group of MDPiHP is present, with a strength of -0.5 kcal/mol. Moreover, compared to the other cathinones, the extended ring system of MDPiHP penetrates further into the protein core of DAT, which might explain its activity. Finally, a slight rotation in the heterocycle could probably allow the formation of the arene-hydrogen interaction with Tyr156, which would add further stability. This conformational rearrangement would require molecular dynamics simulations to be properly assessed. See Fig. 3 for further details.

For 3-Me- and 4-Me-α-PiHP, as well as 3-F- and 4-F-α-PiHP, the interactions with Asp79 and Tyr156 are generally conserved (Figure S1). In these derivatives, substituent positioning within the binding pocket appears to play a key role. In particular, the methyl group in 3-Me-α-PiHP partially occupies the space filled by the benzene ring of cocaine, whereas in 4-Me-α-PiHP it is oriented toward Ser149, where steric constraints are more pronounced, which may account for its lower affinity. A similar trend is observed for the fluorinated analogues, where the reduced potency of 4-F-α-PiHP may be attributed to steric hindrance. In contrast, in 3-F-α-PiHP the fluorine atom is oriented toward a structural water molecule, potentially enabling a stabilizing hydrogen bond.

### α-PiHP analogues produce dose-dependent psychostimulant effects in mice

**Fig. 4:**
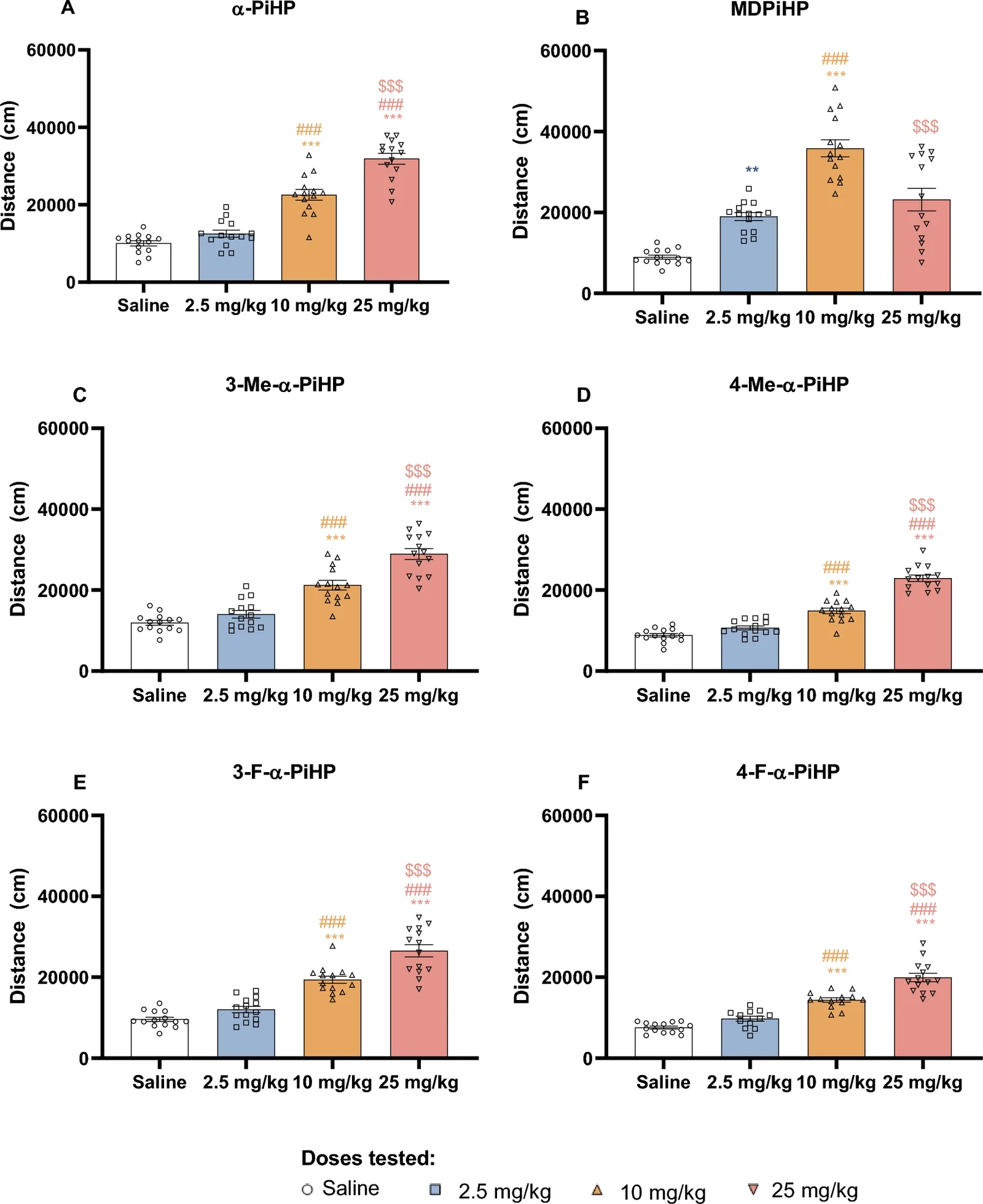
Effects of α-PiHP (**A**) and its derivatives MDPiHP (**B**), 3-Me-α-PiHP (**C**), 4-Me-α-PiHP (**D**), 3-F-α-PiHP (**E**) and 4-F-α-PiHP (**F**) on cumulative locomotor activity in CD-1 male mice. Bars represent mean ± SEM of the distance travelled (in centimetres) during 60 min. Tukey’s multiple comparisons test: **p < 0.01 and ***p < .001 vs. saline; ###*p* < 0.001 vs. 2.5 mg/kg; and $$$*p* < 0.001 vs. 10 mg/kg group. *n* = 13–14/group

As shown in Fig. 4, all compounds produced a significant dose-dependent increase in HLA compared to the saline group, with MDPiHP displaying an inverted U-shaped dose–response curve. Full statistical results are provided in Supplemental Material Table S3. At the intermediate dose (10 mg/kg), MDPiHP was significantly the most effective compound in inducing hyperlocomotion (Figure S2), while at the lowest dose it was the only compound to elicit a significant effect compared with saline.

HLA time courses are shown in Figure S3, and the statistical results of the two-way ANOVA of repeated measures are presented in Supplemental Material Table S3. Following administration of 10 and 25 mg/kg, all compounds produced a rapid onset effect (within 5–10 min).

### α-PiHP analogues induce thigmotaxis at moderate to high doses, except para-substituted derivatives

The time spent in the centre of the OF arena is shown in Figure S4, with the corresponding statistical analyses presented in Supplemental Material Table S3. Except for the *para*-substituted derivatives, a significant decrease in centre occupancy was observed for all cathinones evaluated at 25 mg/kg. At the lowest dose, thigmotaxis was not detected for any of the compounds.

### α-PiHP analogues produce rewarding effects in the CPP test

**Fig. 5:**
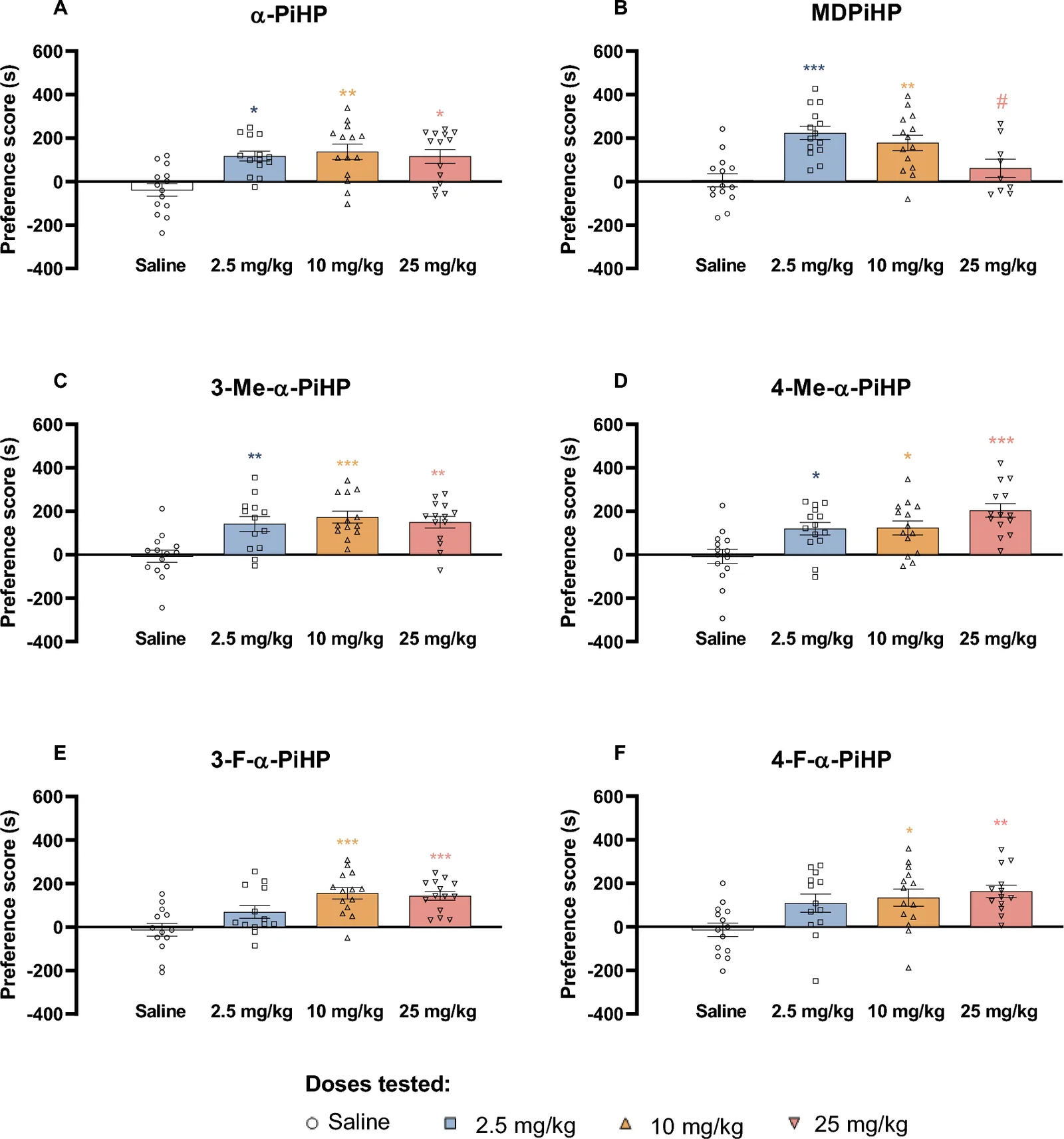
Effects of α-PiHP (**A**) and its derivatives MDPiHP (**B**), 3-Me-α-PiHP (**C**), 4-Me-α-PiHP (**D**), 3-F-α-PiHP (**E**) and 4-F-α-PiHP (**F**) on the CPP test in CD-1 mice. Bars represent mean ± SEM of the preference score (the difference between the time, in seconds, spent in the drug-paired compartment on the test day and the pre-conditioning day). Dunn’s or Tukey’s multiple comparisons test: *p < 0.05, **p < 0.01 and ***p < 0.001 vs. saline; #*p* < 0.05 vs. 2.5 mg/kg group. *n* = 10–14/group

The rewarding effects of α-PiHP and its derivatives were evaluated using the CPP paradigm. 4 animals were withdrawn from the experiments due to an initial preference for one of the compartments (> 70% of the total session time). All compounds significantly increased preference scores at 10 mg/kg, as shown in Fig. 5. At the lowest dose α-PiHP, MDPiHP and methyl substituted compounds (Fig. 5A-D) produced a significant effect compared with saline. However, at the highest dose, MDPiHP was the only compound that did not induce a significant preference score (Fig. 5B). Some fatalities occurred after both acute and/or repeated administrations of MDPiHP (see Supplemental Material Table S4). No fatalities were observed for the other compounds at any dose or regime tested. Full statistical results of the CPP test are provided in Supplemental Material Table S3.

## Discussion

The proliferation of SCs has revealed a niche within the market for cheap and legal stimulants (European Union Drugs Agency 2025), highlighting its potential to become a major player in the stimulants market. Thus, it is crucial to better understand their associated risks to support harm reduction and effective emergency responses. In this context, α-PiHP and its analogues represent a timely model to explore how emerging substitutions within SCs may impact pharmacological activity, abuse liability, and associated public health risks.

The present study provides a systematic characterization of the monoamine transporter inhibitory profile of α-PiHP and its derivatives, revealing a pattern of high dopaminergic and noradrenergic potency combined with comparatively weaker serotonergic activity, resulting in consistently high DAT/SERT ratios. This pharmacological profile is characteristic of pyrrolidinophenone-type cathinones and supports a predominantly stimulant-like mechanism of action (Kolaczynska et al. 2021; Nadal-Gratacós et al. 2022). α-PiHP displayed similar inhibitory potency at hDAT compared to α-PVP. On the other hand, MDPiHP showed greater potency than α-PVP. This pattern suggests that the introduction of a methylenedioxy group confers a clear increase in the affinity for DAT and, consequently, in the inhibitory potency within both the α-PiHP and α-PVP series. In fact, our results demonstrated that the introduction of the methylenedioxy group in the α-PiHP scaffold enables additional stabilizing interactions (notably with Ser149) and promotes deeper penetration of the ligand into the protein core, which may explain such enhanced DAT inhibitory potency and affinity. In line with this, the most potent hDAT inhibitors were MDPV and MDPiHP. Notably, all *meta-*substituted analogues, although non-significantly, showed lower IC_50_ values than their *para*-substituted analogue, which is in line with previous observations by Stalberga et al. 2025. This also agrees with earlier work from our group showing that *meta*-halogen-substituted-α-PVP derivatives are slightly more DAT-selective than the corresponding *para*-substituted analogues (Nadal-Gratacós et al. 2022; Stalberga et al. 2025). This trend does not appear to be limited to halogen substituents, as other studies have also reported increased DAT selectivity for *meta*- versus *para*-ring-substitutions (Cozzi et al. 2013; Walther et al. 2018). Moreover, all *meta-*substituted analogues showed lower *K*_i_ values than its *para*-substituted analogue. The preferential DAT potency of *meta*-substituted analogues over their *para*- counterparts is also structurally supported by the docking data. *Meta*-substitutions are better accommodated within the DAT binding pocket, minimizing steric clashes and, in some cases, enabling additional stabilizing interactions. In contrast, *para*-substituents are oriented toward more sterically constrained regions of the pocket, particularly in the vicinity of Ser149, which plausibly accounts for the consistency lower DAT affinity of 4-substituted derivatives. On the other hand, bulkier substituents have been reported to shift selectivity towards SERT (Bonano et al. 2015; Sakloth et al. 2015; Nadal-Gratacós et al. 2021). Consistent with this notion, although none of the evaluated compounds displayed true SERT selectivity, methyl-substituted cathinones showed a significant increase in SERT affinity compared with unsubstituted or fluorinated derivatives. Moreover, methylenedioxy-substituted compounds exhibited a more pronounced increase in SERT affinity, suggesting that this functional group contributes more effectively to SERT.

Structurally, α-PiHP and MDPiHP differ from α-PVP and MDPV, respectively, by the presence of an additional methyl group in the aliphatic side chain, resulting in a branched isohexyl configuration that enhanced hDAT binding affinity. Moreover, in our study, the *K*_i_ /IC_50_​​ ratios (DAT binding/DA uptake inhibition) for α-PiHP and its analogues were close to unity. According to the criteria established by Eshleman et al. 2017, a ratio near unity (≤ 2) indicates a pure uptake blocker, whereas values substantially greater than unity (typically > 10) are consistent with substrate/releaser activity. Thus, our findings support the classification of α-PiHP and its analogues as monoamine uptake blockers rather than substrates. Furthermore, Timmerman et al. 2026 have recently demonstrated that α-PiHP acts primarily as a monoamine transporter blocker rather than a releaser. However, this observation should be interpreted with caution, as differences in the in vitro methodologies used across studies may limit the direct extrapolation of these ratios to other classes of psychostimulants. Regarding NET inhibition, 4-F-α-PiHP was the least potent compound, and its significantly reduced potency compared to its 3-F-analogue mirrors findings reported by (Stalberga et al. 2025). The remaining α-PiHP derivatives showed slightly greater NET inhibitory potency than α-PiHP itself. While α-PiHP was marginally more potent at DAT than α-PVP, it exhibited reduced NET inhibition relative to α-PVP, which agrees with previous reports from other groups (Persson et al. 2024; Baarset et al. 2025). Although α-PHP, an isomer of α-PiHP, was not included in the present study, earlier reports suggest only minor differences between α-PVP and α-PHP. Eshleman et al. 2017 and Kolaczynska et al. 2021 reported a small decrease in NET and DAT potency with side-chain elongation from α-PVP to α-PHP. However, Kolanos et al. 2015 observed a slight increase in DAT potency for α-PHP. Both compounds have negligible activity at SERT compared to their effects on DAT and NET, but the addition of an extra methyl group in the aliphatic side chain slightly increases its potency. The divergent findings may indicate subtle differences in transporter interaction profiles between α-PiHP and α-PHP despite their isomeric relationship.

α-PiHP has been reported to exert strong psychostimulant effects (Shetty et al. 2023). However, to the best of our knowledge, the psychostimulant profile of the α-PiHP analogues evaluated in the present study has not been previously described. All α-PiHP derivatives tested induced a clear hyperlocomotion response. Notably, MDPiHP, the most potent compound at hDAT, elicited the greatest increase in locomotor activity at the intermediate dose tested (10 mg/kg, i.p.). MDPiHP also showed an inverted U-shaped dose–response curve, which could be explained by the emergence of non-exploratory stereotypies at the highest dose, such as head bobbing and excessive grooming, contributing to the observed reduction in HLA (Baumann et al. 2012). Furthermore, and in agreement with our in vitro findings, 3-Me-α-PiHP induced a higher locomotion response than its *para*-substituted analogue at the intermediate dose. This positional effect of the substituent is consistent with previous results from our research group, in which *meta*-halogen-substituted α-PVP derivatives induced greater hyperlocomotion than their *para*-substituted counterparts (Nadal-Gratacós et al. 2022). Interestingly, whereas *meta-para* differences were clearly observed for the methyl-substituted α-PiHP analogues, these differences were not significant for the fluorinated derivatives (2.5 mg/kg: *p* = 0.3897; 10 mg/kg: *p* = 0.0756; 25 mg/kg: *p* = 0.055). However, the *meta*-fluorinated compound displayed slightly higher locomotor activity than its *para*-substituted counterpart across doses, suggesting a trend that may deserve further investigation.

Anxiety is one of the most frequently reported symptoms in patients admitted to emergency departments following psychostimulant use, and similar anxiety-related effects have also been described after α-PiHP consumption (Liakoni et al. 2015; Harnett et al. 2022; Redona et al. 2022; Balcaen et al. 2025). Rodents naturally display thigmotaxis, or wall-seeking behaviour, when exposed to novel open environments, a phylogenetically conserved response that has been widely used as an indirect measure of anxiety-like behaviour in the open-field test (Simon et al. 1994; Kraeuter et al. 2019). Increased exploration of the central zone is generally interpreted as an anxiolytic-like response, whereas greater preference for the peripheral area is considered indicative of anxiety-like behaviour. Given that all compounds evaluated in the present study exhibited marked DAT inhibition, assessment of thigmotaxis was considered particularly relevant, as increased dopaminergic transmission has previously been associated with enhanced anxiety-like responses and increased wall-seeking behaviour (Simon et al. 1993; Zarrindast and Khakpai 2015). In fact, cocaine, d-amphetamine and methamphetamine, have been shown to increase thigmotaxis, supporting the involvement of dopaminergic mechanisms in this behavioural response (Simon et al. 1994, Struntz et al*.* 2018). Similar behavioural alterations have also been described for synthetic cathinones. Although methylone has been reported to increase exploratory behaviour at certain doses, higher doses have also been associated with reduced time spent in the centre of the arena, suggesting dose-dependent modulation of anxiety-related behaviours (Štefkova et al. 2017). Furthermore, increased thigmotaxis has been described in several pyrrolidine-containing cathinones, further supporting the relevance of this behavioural readout in the context of novel psychoactive substances acting on monoaminergic systems (Nadal-Gratacós et al. 2022, 2025, 2026). In our study, α-PiHP, MDPiHP and both *meta*-substituted α-PiHP derivatives significantly reduced the time spent in the centre of the arena compared with the control group at least at one of the doses tested. Particularly, MDPiHP and 3-F-α-PiHP were the only cathinones to elicit thigmotaxis at the intermediate dose. This observation is particularly relevant given that these compounds also exhibited the highest DAT inhibitory potency in our in vitro assays, further supporting a possible association between enhanced dopaminergic activity and anxiety-like behavioural outcomes. Interestingly, our findings are also consistent with previous observations from structurally related α-pyrrolidinophenones, where *meta*-substituted analogues induced thigmotaxis at lower doses than their *para*-substituted counterparts (Nadal-Gratacós et al. 2022), suggesting that substitution pattern may influence the emergence of anxiety-related effects. Notably, at the highest dose tested, all compounds induced an increase in HLA. Therefore, these behavioural findings should be interpreted cautiously, as locomotor stimulation may partially influence exploratory patterns. It should be noted, however, that thigmotaxis represents a preliminary indicator of anxiety-like behaviour, and that more specific anxiety-related paradigms, such as the elevated plus maze or light–dark box test, would be required to more comprehensively characterize these effects.

Drugs of abuse are known to activate the mesolimbic DA system, producing DA increases that are markedly greater than those elicited by natural rewards, thereby making them highly salient (Pierce and Kumaresan 2006; Baik 2013; Horseman and Meyer 2019). α-PiHP has previously been reported to display abuse potential comparable to that of methamphetamine and cocaine (Shetty et al. 2023). However, to our knowledge, the rewarding properties of its structural derivatives have not been characterized yet. All SCs evaluated in the present study exhibited a strong interaction with hDAT and notably high DAT/SERT ratios, which, as previously mentioned, has been associated with abuse liability (Negus and Banks 2017). In line with these in vitro findings, all tested compounds produced rewarding effects, as evidenced by significant preference scores at least at two of the doses tested. Specifically, α-PiHP, MDPiHP and *methyl*-substituted α-PiHP derivatives induced significant rewarding effects at a dose of 2.5 mg/kg. At this dose, α-PiHP produced rewarding effects comparable to its isomer α-PHP, to α-PVP and to 10 mg/kg of cocaine, which reaffirms that the structural branched isohexyl configuration does not diminish its addictive potential (Gatch et al. 2017; Duart-Castells et al. 2020 and 2021; Shetty et al. 2023). Importantly, at the highest dose tested, MDPiHP was the only cathinone that did not produce a statistically significant rewarding effect, despite a still positive preference score. The reduction in locomotor activity due to the appearance of stereotypies may reflect strong adverse effects that induces such a loss of significant drug reward, a phenomenon previously observed with other cathinones (Gatch et al. 2017; Nadal-Gratacós et al. 2021 and 2023).

As mentioned previously, numerous cases of severe intoxication, including fatal outcomes, have been reported following cathinone use (Beck et al. 2018; Zaami et al. 2018; Pieprzyca et al. 2022; Wachholz et al. 2023; Balcaen et al. 2025; Macháček and Bébarová 2026). This highlights the importance of thoroughly characterizing the pharmacological and toxicological profiles of these compounds. Notably, MDPiHP caused lethality at the highest dose tested at various stages of exposure, underscoring substantial toxicity. In contrast, such lethality was not observed for the other compounds evaluated under the same experimental conditions.

Finally, while this study provides robust evidence of the SAR of α-PiHP derivatives in male mice, we acknowledge the absence of female subjects as a limitation. Male mice were selected to ensure comparability with previous studies on synthetic cathinones, to minimize hormonal variability in these initial screening stages and considering the present study as a preliminary characterization of novel α-PiHP analogues to establish their basic pharmacological profile. Nevertheless, future research exploring potential sex differences in the effects of these compounds is warranted.

## Conclusion

In conclusion, the present study provides an integrated in silico, in vitro, and in vivo characterization of α-PiHP and a series of structurally related analogues, highlighting how relatively small chemical modifications can markedly influence monoamine transporter interactions, behavioural outcomes, and overall risk profiles. All compounds displayed a predominantly stimulant-like pharmacological signature, characterized by high DAT and NET reuptake inhibition and elevated DAT/SERT ratios, which translated into robust psychostimulant activity and rewarding effects in vivo*.* Positional isomerism emerged as a key determinant of activity, with *meta*-substituted analogues generally exhibiting greater DAT affinity and selectivity, as well as behavioural stimulation than their *para*-substituted counterparts, a trend supported by molecular docking analyses. Among the compounds studied, MDPiHP stood out due to its high DAT potency, pronounced behavioural effects, and notable lethality rate, underscoring the potential for severe toxicity even following limited exposure. Taken together, these findings reinforce the significant abuse liability and health risks associated with emerging α-pyrrolidinophenone cathinones and emphasize the importance of systematic pharmacological evaluation to inform harm reduction strategies, clinical management of intoxications, and early risk assessment of newly emerging NPS.

## Supplementary Information

Below is the link to the electronic supplementary material.

Supplementary file1 (DOCX 2362 kb)

## Data Availability

Data will be made available on request.
