# Supplementary material for: Structure–activity relationship of synthetic cathinones: integrated in silico, in vitro, and in vivo studies of α-PiHP analogues
Source: Arch Toxicol. 2026 Jun 27;100(11):5165–80. doi: 10.1007/s00204-026-04483-z (PMC13694374; doi:10.1007/s00204-026-04483-z)
Supplement: Supplementary file 1 [file 204_2026_4483_MOESM1_ESM.docx]

**Structure–Activity Relationships of Synthetic Cathinones:** I**ntegrated *In Silico*, *In Vitro*, and *In Vivo* Studies of α-PiHP Analogues**

Martalu D. Pazos¹, Guillermo García-Díez^2^, David Pubill^1^, Xavier Berzosa^2^, Roger Estrada-Tejedor^3^, Jorge Camarasa^1^, Elena Escubedo^1^, Raúl López-Arnau^1*^ and Núria Nadal-Gratacós^1*^

¹ Department of Pharmacology, Toxicology and Therapeutic Chemistry, Pharmacology Section, Institute of Biomedicine (IBUB), Faculty of Pharmacy and Food Science, Universitat de Barcelona, 08028 Barcelona, Spain

² Chemical Reactions for Innovative Solutions (CRISOL), IQS School of Engineering, Universitat Ramon Llull, 08017 Barcelona, Spain

^3^ Department of Pharmaceutical Chemistry, IQS School of Engineering, Universitat Ramon Llull, 08017 Barcelona, Spain

**Synthesis of α-PiHP and its derivatives**

For the synthesis of α-PiHP, a starting benzonitrile was subjected to a reaction with a Grignard reagent (3-methylbutylmagnesium bromide) in anhydrous conditions followed by acidic hydrolysis (10% H_2_SO_4_), to achieve the intermediate ketone with the characteristic isopropyl group of this molecule. For the α-halogenation, the ketone was dissolved in dichloromethane (CH_2_Cl_2_) under acidic conditions [catalytic amounts of acetic acid (AcOH)] followed by the addition of bromine (Br_2_). To obtain the final pyrovalerone, a nucleophilic substitution was performed by adding pyrrolidine in ethanol, followed by the dropwise addition of HCl (3 M) in cyclopentyl methyl ether to induce crystallization of the corresponding salt.

For the synthesis of the ring-substituted α-PiHP derivatives, the initial benzonitrile was substituted with a methyl or fluoro group in the meta-position to obtain 3-Me-α-PiHP and 3-F-α-PiHP, or in the para-position to obtain 4-Me-α-PiHP and 4-F-α-PiHP; MDPiHP was obtained using piperonylonitrile.

**Scheme S1**. Synthesis of α-PiHP analogues

The structure of the six synthesized pyrovalerones was verified by proton (^1^H) and carbon (^13^C) nuclear magnetic resonance (NMR), yielding the following results:

MDPiHP (39% yield):

^1^H NMR (400 MHz, CDCl_3_) δ 12.16 (s, 1H), 7.64 (d, *J* = 8.9 Hz, 1H), 7.43 (s, 1H), 6.92 (d, *J* = 8.1 Hz, 1H), 6.09 (s, 2H), 5.14 (s, 1H), 3.81 (s, 2H), 3.59 (s, 1H), 2.81 (s, 1H), 2.23 – 2.08 (m, 3H), 2.04 (d, *J* = 10.7 Hz, 4H), 1.92 (dd, *J* = 13.8, 5.4 Hz, 1H), 1.73 – 1.61 (m, 1H), 0.96 (d, *J* = 6.6 Hz, 3H), 0.94 (d, *J* = 6.5 Hz, 3H). ^13^C NMR (100 MHz, CDCl3) δ 194.36, 153.60, 148.92, 130.63, 125.73, 108.37, 107.63, 102.32, 59.54, 52.66, 48.40, 39.65, 24.69, 23.67, 23.28, 22.91, 21.99.

4-Me-α-PiHP (38% yield):

^1^H NMR (400 MHz, CDCl_3_) δ 12.42 (s, 1H), 7.87 (d, J = 8.4 Hz, 2H), 7.33 (d, J = 7.9 Hz, 2H), 5.18 (t, J = 4.3 Hz, 1H), 3.83 (d, J = 7.0 Hz, 2H), 3.63 – 3.55 (m, 1H), 2.85 – 2.73 (m, 1H), 2.43 (s, 3H), 2.21 – 2.10 (m, 2H), 2.09 – 2.00 (m, 2H), 1.91 (t, J = 11.6 Hz, 2H), 1.70 (dd, J = 12.9, 8.0 Hz, 1H), 0.95 (d, J = 2.7 Hz, 3H), 0.94 (d, J = 2.5 Hz, 3H). ^13^C NMR (100 MHz, CDCl3) δ 196.36 146.70, 133.43, 130.17, 128.70, 59.76, 52.91, 48.45, 39.79, 24.84, 23.90, 23.53, 22.99, 22.20, 21.81.

3-F-α-PiHP (44% yield):

^1^H NMR (400 MHz, CDCl_3_) δ 12.49 (s, 1H), 7.83 (d, *J* = 7.9 Hz, 1H), 7.68 (d, *J* = 11.9 Hz, 1H), 7.59 – 7.51 (m, 1H), 7.42 – 7.35 (m, 1H), 5.26 (s, 1H), 3.92 – 3.57 (m, 4H), 2.85 (s, 1H), 2.34 (s, 1H), 2.18 (d, *J* = 12.4 Hz, 3H), 2.10 – 1.95 (m, 3H), 1.94 – 1.85 (m, 1H), 1.78 – 1.69 (m, 1H), 0.97 (s, 3H), 0.95 (s, 3H). ^13^C NMR (100 MHz, CDCl3) δ 196.53, 164.79, 162.30, 138.25, 131.72, 125.02, 122.86, 122.65, 115.83, 115.60, 61.10, 53.46, 49.38, 40.18, 25.32, 24.41, 24.02, 23.43, 22.68.

α-PiHP (32% yield):

^1^H NMR (400 MHz, CDCl_3_) δ 12.59 (s, 1H), 7.97 (d, J = 8.4 Hz, 2H), 7.70 (t, J = 6.9 Hz, 1H), 7.56 (t, J = 7.7 Hz, 2H), 5.20 (t, J = 8.9 Hz, 1H), 3.84 (dt, J = 15.2, 8.8 Hz, 2H), 3.62 (dq, J = 11.6, 6.1 Hz, 1H), 2.85 – 2.73 (m, 1H), 2.26 – 2.16 (m, 2H), 2.12 – 2.04 (m, 2H), 2.03 – 1.96 (m, 1H), 1.95 – 1.86 (m, 1H), 1.77 (dq, J = 14.1, 6.9 Hz, 1H), 0.97 (dd, J = 6.4, 2.3 Hz, 6H). ^13^C NMR (100 MHz, CDCl_3_) δ 197.10, 136.01, 135.40, 129.68, 128.70, 59.90, 53.16, 48.49, 39.97, 25.03, 24.15, 23.78, 23.09, 22.42.

3-Me-α-PiHP (36% yield):

^1^H NMR (400 MHz, CDCl_3_) δ 12.54 (s, 1H), 7.75 (d, J = 7.6 Hz, 2H), 7.51 (d, J = 7.2 Hz, 1H), 7.48 – 7.40 (m, 1H), 5.15 (ddd, J = 9.1, 5.9, 3.6 Hz, 1H), 3.92 – 3.76 (m, 2H), 3.68 – 3.55 (m, 1H), 2.77 (dq, J = 10.5, 7.4 Hz, 1H), 2.45 (s, 3H), 2.26 – 2.14 (m, 2H), 2.08 (ddd, J = 13.7, 7.8, 6.0 Hz, 2H), 2.04 – 1.86 (m, 2H), 1.79 – 1.73 (m, 1H), 0.97 (d, J = 6.6 Hz, 6H). ^13^C NMR (100 MHz, CDCl_3_) δ 197.22, 139.73, 136.25, 136.04, 129.58, 129.38, 126.15, 60.33, 53.51, 48.87, 40.00, 25.01, 23.90, 23.20, 22.56, 21.60.

4-F-α-PiHP (40% yield):

^1^H NMR (400 MHz, CDCl_3_) δ 12.33 (s, 1H), 8.11 (dd, *J* = 8.9, 5.2 Hz, 2H), 7.25 – 7.15 (m, 2H), 5.39 (ddd, *J* = 8.4, 5.8, 4.0 Hz, 1H), 3.90 (ddt, *J* = 10.6, 7.2, 5.4 Hz, 1H), 3.76 (dq, *J* = 10.5, 7.4 Hz, 1H), 3.61 (tt, *J* = 11.5, 6.2 Hz, 1H), 2.88 (dq, *J* = 10.8, 7.6 Hz, 1H), 2.27 – 2.14 (m, 2H), 2.13 – 1.94 (m, 3H), 1.88 (ddd, *J* = 13.6, 8.4, 6.1 Hz, 1H), 1.69 (ddq, *J* = 12.8, 7.9, 6.4 Hz, 1H), 0.94 (dd, *J* = 6.5, 2.1 Hz, 6H). ^13^C NMR (100 MHz, CDCl_3_) δ 195.76, 168.25, 165.67, 132.45, 132.42, 131.86, 131.76, 116.95, 116.73, 60.68, 53.04, 49.12, 39.87, 24.98, 24.04, 23.65, 23.16, 22.32.

**Figure S1:** Binding interactions of 3-Me-α-PiHP (red, **A**), 4-Me-α-PiHP (yellow, **B**) and 4-F-α-PiHP (grey, **C**) with DAT.

**Figure S2:** Comparison of the effects of α-PiHP and its derivatives across doses on cumulative locomotor activity at 2.5 mg/kg **(A)**, 10 mg/kg **(B)** and 25 mg/kg **(C)** in CD-1 mice. Bars represent mean ± SEM of the distance travelled (in centimetres) during 60 min. Tukey’s multiple comparisons test: ***p< 0.01 and ***p< 0.001 vs. α-PiHP; ###p< 0.001 vs. MDPiHP and $$p< 0.01 vs. 3-Me-α-PiHP. n* = 13-14/group.

**Figure S3:** Time course profile of HLA induced by α-PiHP **(A)**, MDPiHP **(B)**, 3-Me-α-PiHP **(C)**, 4-Me-α-PiHP **(D)**, 3-F-α-PiHP **(E)** and 4-F-α-PiHP **(F).** Each time point represents the mean ± SEM of the distance (in centimetres) travelled in 5-minute blocks. Only comparisons vs the corresponding saline group are shown for clarity purposes. Tukey’s multiple-comparisons test: **p< 0.05, **p< 0.01 and ***p< 0.001 vs. saline group. n* = 13-14/group.

**Figure S4:** Effects of α-PiHP **(A)**, MDPiHP **(B)**, 3-Me-α-PiHP **(C)**, 4-Me-α-PiHP **(D)**, 3-F-α-PiHP **(E)** and 4-F-α-PiHP **(F)** (2.5, 10 and 25 mg/kg, i.p.) on the Open Field test in CD-1 male mice. Bars represent mean ± SEM of time in the centre. Dunn's or Tukey’s multiple comparisons test: ** p< 0.05 and *** p< 0.001 vs. saline, # p< 0.05 and ### p< 0.001 vs. 2.5 mg/kg and $$p< 0.01 vs. 10 mg/kg. n* = 12-14/group.

**Table S1.** Statistical results of the IC_50_ values for the uptake inhibition assay at hNET, hDAT and hSERT (One-way ANOVA) and of the *K*_i_ values for the binding assay at hDAT (One-way ANOVA).

| **Monoamine**  **transporter** | **Assay** | **Statistical results** | | | | |
| --- | --- | --- | --- | --- | --- | --- |
|  |  | **F (DFn, DFd)/**  **H(DFn)** | ***p*** | **Variables** | | ***p*** |
| **hNET** | **Uptake**  **Inhibition** | H (7) =21,10 | *p<*0.01 | 4-F-α-PiHP | 3-F-α-PiHP | *p<*0.1 |
|  |  |  |  |  | MDPiHP | *p<*0.1 |
| **hDAT** |  | F (7, 24) = 19,52 | *p<*0.001 | α-PVP | 4-F-α-PiHP | *p<*0.001 |
|  |  |  |  |  | 4-Me-α-PiHP | *p<*0.001 |
|  |  |  |  |  | 3-Me-α-PiHP | *p<*0.001 |
|  |  |  |  |  | MDPV | *p<*0.001 |
|  |  |  |  |  | 3-F-α-PiHP | *p<*0.001 |
|  |  |  |  |  | MDPiHP | *p<*0.001 |
|  |  |  |  | α-PiHP | 4-Me-α-PiHP | *p<*0.01 |
|  |  |  |  |  | 3-Me-α-PiHP | *p<*0.01 |
|  |  |  |  |  | MDPV | *p<*0.001 |
|  |  |  |  |  | 3-F-α-PiHP | *p<*0.001 |
|  |  |  |  |  | MDPiHP | *p<*0.001 |
| **hSERT** |  | F (7, 24) = 121,2 | *p<*0.001 | α-PVP | α-PiHP | *p<*0.001 |
|  |  |  |  |  | 3-F-α-PiHP | *p<*0.001 |
|  |  |  |  |  | 4-F-α-PiHP | *p<*0.001 |
|  |  |  |  |  | 3-Me-α-PiHP | *p<*0.001 |
|  |  |  |  |  | 4-Me-α-PiHP | *p<*0.001 |
|  |  |  |  |  | MDPV | *p<*0.001 |
|  |  |  |  |  | MDPiHP | *p<*0.001 |
|  |  |  |  | α-PiHP | 3-F-α-PiHP | *p<*0.001 |
|  |  |  |  |  | 4-F-α-PiHP | *p<*0.001 |
|  |  |  |  |  | 3-Me-α-PiHP | *p<*0.001 |
|  |  |  |  |  | 4-Me-α-PiHP | *p<*0.001 |
|  |  |  |  |  | MDPV | *p<*0.001 |
|  |  |  |  |  | MDPiHP | *p<*0.001 |
|  |  |  |  | 3-F-α-PiHP | 3-Me-α-PiHP | *p<*0.01 |
|  |  |  |  |  | 4-Me-α-PiHP | *p<*0.001 |
|  |  |  |  |  | MDPV | *p<*0.001 |
|  |  |  |  |  | MDPiHP | *p<*0.001 |
|  |  |  |  | 4-F-α-PiHP | 3-Me-α-PiHP | *p<*0.01 |
|  |  |  |  |  | 4-Me-α-PiHP | *p<*0.01 |
|  |  |  |  |  | MDPV | *p<*0.001 |
|  |  |  |  |  | MDPiHP | *p<*0.001 |
| **hDAT** | **Binding** | F (7, 25) = 12,55 | *p<*0.001 | 4-Me-α-PiHP | 3-F-α-PiHP | *p<*0.1 |
|  |  |  |  |  | 3-Me-α-PiHP | *p<*0.01 |
|  |  |  |  |  | MDPV | *p<*0.01 |
|  |  |  |  |  | α-PiHP | *p<*0.01 |
|  |  |  |  |  | MDPiHP | *p<*0.001 |
|  |  |  |  | 4-F-α-PiHP | 3-F-α-PiHP | *p<*0.1 |
|  |  |  |  |  | 3-Me-α-PiHP | *p<*0.01 |
|  |  |  |  |  | MDPV | *p<*0.01 |
|  |  |  |  |  | α-PiHP | *p<*0.01 |
|  |  |  |  |  | MDPiHP | *p<*0.001 |
|  |  |  |  | α-PVP | α-PiHP | *p<*0.1 |
|  |  |  |  |  | MDPiHP | *p<*0.001 |

**Table S2**: London dG Scoring Values versus *K*_i_ values for each studied cathinone.

| **Molecule** | ***K*_i_ (nM)** | **London dG Scoring Value** |
| --- | --- | --- |
| MDPiHP | 3.00 | -12.33 |
| α-PiHP | 8.57 | -11.05 |
| 3-Me-α-PiHP | 10.21 | -11.55 |
| 3-F-α-PiHP | 12.06 | -11.42 |
| 4-F-α-PiHP | 24.24 | -11.45 |
| 4-Me-α-PiHP | 24.61 | -11.74 |

|  |  | **Statistical results** | | | | | | | |
| --- | --- | --- | --- | --- | --- | --- | --- | --- | --- |
|  |  | **HLA** | | **HLA time course** | | **OF** | | **CPP** | |
| **Drug** | **Variable** | **F (DFn, DFd)** | ***p*** | **F (DFn, DFd)** | ***p*** | **F (DFn, DFd)/ H(DFn)** | ***p*** | **F (DFn, DFd)/ H(DFn)** | ***p*** |
| α-PiHP | Dose | F (3, 52) = 76,18 | *p<*0.001 | F (3, 52) = 76,18 | *p<*0.001 | F (3, 50) = 11,12 | *p<*0.001 | H (3) =15,00 | *p<*0.001 |
|  | Time | N/A | | F (11, 572) = 123,8 | *p<*0.001 | N/A | | N/A | |
|  | Interaction |  |  | F (33, 572) = 7,559 | *p<*0.001 |  |  |  |  |
| 3-Me-α-PiHP | Dose | F (3, 52) = 52,80 | *p<*0.001 | F (3, 52) = 53,27 | *p<*0.001 | F (3, 51) = 22,24 | *p<*0.001 | F (3, 50) = 8,115 | *p<*0.001 |
|  | Time | N/A | | F (11, 569) = 93,21 | *p<*0.001 | N/A | | N/A | |
|  | Interaction |  |  | F (33, 569) = 5,657 | *p<*0.001 |  |  |  |  |
| 4-Me-α-PiHP | Dose | F (3, 52) = 101,2 | *p<*0.001 | F (3, 52) = 104,2 | *p<*0.001 | H (3) = 1,98 | p>0.1 | F (3, 52) = 7,891 | *p<*0.001 |
|  | Time | N/A | | F (11, 565) = 127,9 | *p<*0.001 | N/A | | N/A | |
|  | Interaction |  |  | F (33, 565) = 12,70 | *p<*0.001 |  |  |  |  |
| 3-F-α-PiHP | Dose | F (3, 52) = 58,35 | *p<*0.001 | F (3, 52) = 59,01 | *p<*0.001 | H (3) = 16,96 | *p<*0.001 | F (3, 50) = 8,640 | *p<*0.001 |
|  | Time | N/A | | F (11, 567) = 70,15 | *p<*0.001 | N/A | | N/A | |
|  | Interaction |  |  | F (33, 567) = 4,792 | *p<*0.001 |  |  |  |  |
| 4-F-α-PiHP | Dose | F (3, 49) = 62,65 | *p<*0.001 | F (3, 49) = 58,09 | *p<*0.001 | H (3) = 9,96 | *p<*0.1 | F (3, 50) = 4,899 | *p<*0.01 |
|  | Time | N/A | | F (11, 533) = 94,12 | *p<*0.001 | N/A | | N/A | |
|  | Interaction |  |  | F (33, 533) = 6,166 | *p<*0.001 |  |  |  |  |
| MDPiHP | Dose | F (3, 52) = 36,07 | *p<*0.001 | F (3, 52) = 37,10 | *p<*0.001 | F (3, 46) = 10,05 | *p<*0.001 | F (3, 47) = 9,321 | *p<*0.001 |
|  | Time | N/A | | F (11, 540) = 16,33 | *p<*0.001 | N/A | | N/A | |
|  | Interaction |  |  | F (33, 540) = 5,299 | *p<*0.001 |  |  |  |  |

**Table S3.** Statistical results of HLA (One-way ANOVA), HLA time course (Two-way ANOVA of repeated measures), OF (One-way ANOVA) and CPP (One-way ANOVA).

| **MDPiHP** | | |
| --- | --- | --- |
| **Number of exposures to the 25 mg/kg dose** | **Mice** | **Lethality (%)** |
| 1^st^->HLA | 2/14 | 14,29 |
| 2^nd^->CPP | 1/12 | 8,33 |
| 3^rd^->CPP | 0 | 0 |
| 4^th^->CPP | 1/11 | 9,09 |
| 5^th^->CPP | 0 | 0 |

**Table S4**: Lethality rate of MDPiHP at the highest dose.
